# EPI proton resonant frequency temperature mapping at 0.5T in the brain: Comparison to single‐echo gradient recalled echo

**DOI:** 10.1002/mrm.30373

**Published:** 2024-11-11

**Authors:** Diego F. Martinez, Curtis N. Wiens, Chad T. Harris, William B. Handler, Blaine A. Chronik

**Affiliations:** ^1^ The xMR Labs, Department of Physics and Astronomy Western University London Ontario Canada; ^2^ Research and Development Synaptive Medical Toronto Ontario Canada

**Keywords:** Echo planar imaging, gradient recalled Echo, magnetic resonance imaging, MRI phantom, temperature mapping, thermometry

## Abstract

**Purpose:**

Evaluate the use of both single‐echo gradient recalled echo (SE‐GRE) and EPI approaches to creating temperature maps on a mid‐field head‐only scanner, both in vivo and on a tissue mimicking gel.

**Methods:**

Three 2D protocols were investigated (an SE‐GRE, single‐shot EPI, and an averaged single‐shot EPI). The protocols used either a gradient recalled acquisition or an echo planar acquisition, with EPI parameters optimized for the longer $T_{2}^{*}$ at lower field‐strengths. Phantom experiments were conducted to evaluate temperature tracking while cooling, comparing protocol to measurements from an optical fiber thermometer. Studies were performed on a 0.5T head only MR scanner. Temperature stability maps were produced in vivo for the various protocols to evaluate precision.

**Results:**

The use of an EPI protocol for thermometry improved temperature precision in a temperature control phantom and provided an 18% improvement in temperature measurement precision in vivo. Temperature tracking using a fast (<2 s) update rate EPI thermometry sequence provided a similar precision to the slower SE‐GRE protocol.

**Conclusion:**

While SE‐GRE PRF thermometry shows good performance, EPI methods offer improved tracking precision or update rate, making them a better option for thermometry in the brain at mid‐field.

## 1 INTRODUCTION

Thermal therapies are an established clinical treatment protocol for a variety of ailments and refer to the medical application of heat or cold to the body for therapeutic purposes.^1^, ^2^ Heat therapy, also known as hyperthermia treatment, involves the use of various energy sources, such as radiofrequency, laser, microwave, or ultrasound, to generate controlled heat as a common treatment for a variety of ailments.^3^, ^4^, ^5^, ^6^ These minimally invasive techniques offer several advantages, including a reduced risk of complications, shorter recovery time, and potential for outpatient treatment, making it a valuable alternative to invasive traditional surgical interventions.

MR thermometry can be used to non‐invasively track temperatures and has been shown to measure temperatures in ranges between 20°C to 100°C.^1^ In measuring temperature, various tissue parameters have been explored as candidates, including T_1_ change, apparent diffusion coefficient change, magnetization transfer, and apparent proton density imaging.^2^, ^7^, ^8^, ^9^ However, most studies which track thermal therapies have used the proton resonant frequency shift (PRF) method,^8^, ^10^, ^11^ now the preferred method for thermometry. By measuring the difference in sequential MR phase images from a gradient echo acquisition, a temperature map can be determined based on the known relation between temperature and resonant frequency.^12^

Small footprint mid‐field (0.3 to 1.0T)^13^ systems offer unique possibilities for monitoring of interventional procedures.^14^ The compact design and fringe field allow for installation at or near the point‐of‐care. Reduced field strength provides lower susceptibility‐based geometric distortion and reduced specific absorption rate (SAR)‐based tissue heating.^15^, ^16^, ^17^ These are particularly relevant in populations with implantable medical devices, which can be precluded from receiving MR scans at higher field strength.^18^

PRF‐based MR thermometry at mid‐field poses unique challenges. Signal‐to‐noise at this field strength is reduced due to the reduction in polarization. Furthermore, the change in frequency for a fixed change in temperature is proportional to main field strength. This slower phase accrual implies that long TEs are required to generate sufficient phase changes. Fortunately, due to the long $T_{2}^{*}$ at this field strength [at 0.55T: white matter = 72.1 ms, gray matter = 86.4],^19^ longer TEs can be used with reduced signal degradation. However, imaging using longer TEs with traditional single‐echo gradient recalled echo (SE‐GRE) acquisitions will result in reduced spatial coverage, spatial resolution, or image update rate.

EPI‐based thermometry could potentially address the limitations of SE‐GRE acquisitions in the mid‐field. The efficient EPI readout train allows for fast update rates at clinically relevant spatial resolutions (˜2 mm in‐plane).^20^ Optimal TEs (TE = $T_{2}^{*}$) can leverage the longer $T_{2}^{*}$ at mid‐field to improve temperature sensitivity.^21^ The reduced bandwidth in the phase encode direction makes EPI prone to image distortion. However, as mentioned previously, susceptibility induced field inhomogeneities are substantially reduced at this field strength.^15^, ^18^ This suggests EPI acquisitions are a promising alternative to SE‐GRE at this field strength.

The purpose of this study was to evaluate the performance of PRF‐based MR thermometry using SE‐GRE and EPI acquisitions at 0.5T. Using optical temperature probes as a reference, temperature tracking experiments were performed to evaluate the precision and accuracy of both acquisition methods in a phantom. In addition, temperature stability was assessed in‐vivo to confirm clinical viability.

## 2 METHODS

Phantom and in‐vivo experiments were performed using three protocols. The first protocol was a SE‐GRE with a 7.8 s update rate. The second was an averaged single‐shot EPI (ss‐EPI) acquisition with spatial coverage, spatial resolution, and temporal resolution closely matching that of the single echo gradient echo reference protocol (7.6 s update rate). The third was a single‐shot EPI acquisition with faster update rate (1.5 s) and improved spatial coverage. EPI protocols were optimized for thermometry performance in brain tissue.^22^ Detailed imaging parameters for each protocol are given in Table 1. In‐vivo experiments were acquired on a single healthy volunteer with informed consent in compliance with health and safety protocols. All imaging was acquired on a head‐specific 0.5T MR system (Synaptive Medical, Toronto, Ontario) equipped with an eight‐channel head coil and gradients capable of 100 mT/m peak strength and 400 T/m/s slew rate for each axis. For all acquisitions, channel combination was performed using an external calibration pre‐scan in combination with an image‐space variant of the direct virtual coil method.^23^

**TABLE 1:** Parameters for the three sequences used in the temperature stability and temperature tracking tests.

Parameter	SE‐GRE	Averaged single‐shot EPI	Single‐shot EPI
TE (ms)	24	70	70
TR (ms)	86	346	1508
N_avg_	1	22	1
Update rate (s)	7.8	7.6	1.5
Readout bandwidth (kHz)	20	90	90
FOV (mm)	230 × 190	230 × 190	230 × 190
Slices	3	3	13
Slice thickness (mm)	3.0	3.0	3.0
Matrix size	110 × 90	110 × 90	110 × 90
Echo train length	–	1	1
Flip angle (°)	28	54	85

*Note*: Twenty‐two averages were taken on the three‐slice EPI acquisition to achieve a similar update rate to the SE‐GRE sequence. Flip angle was determined using the Ernst angle for the TR of each protocol.

### 2.1 Phantom production

A custom double nested cylinder was produced, with the outer layer acting as a reference region, and the inner cylinder resting in polyurethane foam susceptibility matched using Pyrolytic Graphite.^24^ The outer cylinder has a diameter of 12.7 cm, with inner diameter of 8.97 cm and a length of 10.7 cm. The inner sample container was a 120 mL tube, with length of 7 cm and diameter of 5 cm.

The susceptibility matching foam was created by dispersing pyrolytic graphite at a volume fraction of 0.045 to match tissue susceptibility of −9 ppm into a one component of a two‐component polyurethane foam (Smooth‐On, Macungie, Pennsylvania, USA). This foam was molded to the shape of the sample container. In both the sample and the outer cylinder, a probe insert was made to allow access for an optical temperature probe to be placed in either compartment.

The inner and outer cylinders of the phantom were filled with a hydroxyethylcellulose (HEC) tissue mimicking gel doped with 2 mM copper (II) sulfate.^25^ This gel has T_1_ = 659 ms, T_2_ = 127 ms, and a PRF alpha parameter of −0.00874 ppm/°C.

### 2.2 Temperature tracking experiment

To evaluate the temperature estimation accuracy, the inner cylinder of the phantom described above was heated in a hot water bath to ˜60°C and equipped with a fiber optic temperature probe (LSENS‐T; Rugged Monitoring, Quebec, CA). For all three protocols, 30 min of imaging data were collected as the phantom cooled to room temperature. Temperature maps were computed by taking the phase difference of each time point with respect to the first image of the series. A first‐order polynomial fit was performed over the reference region of the phantom (outer cylinder) and used to remove baseline phase changes due to scanner and environmental instabilities.^12^ Phase differences were scaled to temperature using the PRF alpha parameter and the values over a 1 × 1 cm^2^ region near the probe location were averaged together. Bland–Altman plots were produced for each of the protocol methods to evaluate the measurement bias between the PRF temperature measurements and temperature measured using the fiber optic probe. The SDs of temperature time series in the reference region voxels were used to determine a precision on PRF temperature measurement. These were compared between the SE‐GRE and averaged ss‐EPI protocols using a Welch's t‐test.

### 2.3 In‐vivo temperature stability measurement

In‐vivo temperature stability measurements were performed for all protocols shown in Table 1. Volunteer imaging was conducted under informed consent and in compliance with health and safety protocols at Synaptive Medical Inc. For each protocol, 64 time points were acquired. For each image set, as described above, the phase difference of each image was calculated with respect to the first time point. A first‐order polynomial fit of a reference region on the boundary of the brain was used to remove baseline phase changes^12^ (an example of this region is shown in Figure 4). The SD and the mean average error (MAE) in the temperature time series for each voxel was computed to estimate and compare temperature precisions. The distributions of MAE for the SE‐GRE and single‐shot averaged EPI were compared using an unpaired Welch's t‐test to determine whether any improvements were significant.

## 3 RESULTS

**FIGURE 1:**
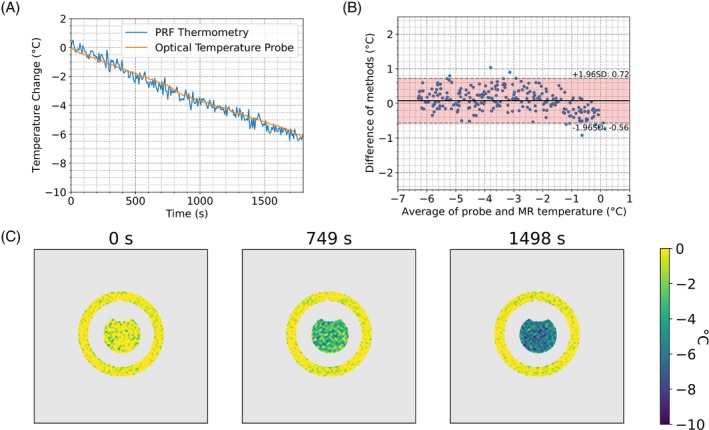
(A) Relative temperature change using a SE‐GRE PRF sequence over a 1 × 1 cm^2^ region (blue) and a fiber optic temperature probe found near the ROI (orange), and (B) a Bland–Altman plot of the bias in temperature tracking between the two temperature measurement methods. Measurements conducted on a custom HEC gel temperature phantom. SE parameters shown in Table 1, with an update rate of 7.8 s. SD while cooling was measured on the outer reference cylinder, with average SD of 0.57°C. (C) Temperature mapping scans at three timepoints during cooling.

**FIGURE 2:**
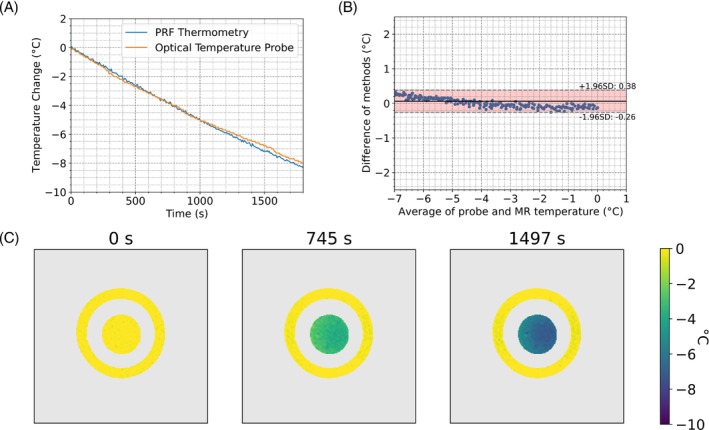
(A) Relative temperature change using an averaged SS‐EPI PRF sequence over a 1 × 1 cm^2^ region with parameters listed in Table 1 (blue) and a fiber optic temperature probe found near the ROI (orange), and (B) a Bland–Altman plot of the temperature bias and agreement between the two tracking methods. Measurements conducted on a custom HEC gel temperature phantom. Twenty‐two averages taken in the SS‐EPI acquisition to achieve a similar update rate as the SE‐GRE protocol. The average SD of the voxels in the reference region over the cooling experiment was 0.14°C. (C) Temperature maps acquired at three time points while cooling.

**FIGURE 3:**
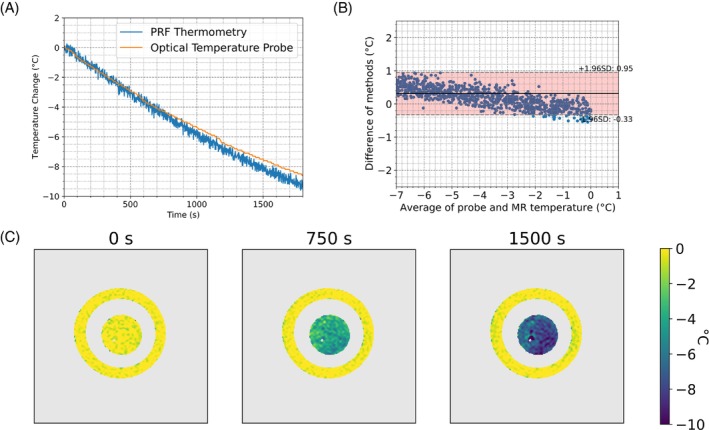
(A) Relative temperature change using a SS‐EPI PRF sequence over a 1 × 1 cm^2^ region with parameters listed in Table 1 (blue) and a fiber optic temperature probe found near the ROI (orange), and (B) a Bland–Altman plot of the temperature variations. Update rate of 1.5 s, with no averaging of time points. SD measured on the outer reference band while cooling averaged 0.34°C. (C) sample timepoints of temperature scans are shown.

The temperature of the thermal phantom was tracked over 30 min using the SE‐GRE acquisition. Temperature measurements from PRF and optical temperature probe show the temporal dynamics of the cooling phantom (Figure 1A). Bland–Altman plots calculate the temperature measurement bias between the two temperature methods to be 0.08°C with a 95% range of 1.28°C (Figure 1B). Representative temperatures maps are shown at times 0 s, 749 s, and 1498s (Figure 1C). Using the outer ring used as reference, the time series SD gave a measure of the temperature measurement precision, with an average SD in the outer ring of 0.56°C and SD on this average of 0.05°C (*N* = 3222 voxels). Matching temperature tracking experiments were performed using the averaged ss‐EPI (Figure 2) and ss‐EPI (Figure 3) acquisitions. The averaged ss‐EPI had a temperature measurement bias of 0.06°C, with 95% spread of 0.64°C. The average SD in the outer ring during the experiment was 0.14°C with a SD on this measure of 0.04°C (*N* = 3692 voxels). An unpaired Welch's t‐test between the SE‐GRE and averaged ss‐EPI showed a statistically significant difference in measurement precision between these methods (*p* < 0.0001).

The Bland–Altman plot on the ss‐EPI acquisition showed a measurement bias of 0.32°C, with a 95% spread of 1.28°C. The temperature measurement precision analysis on the outer ring reported an average voxel time series SD of 0.34°C with a SD of 0.05°C (*N* = 4006).

**FIGURE 4:**
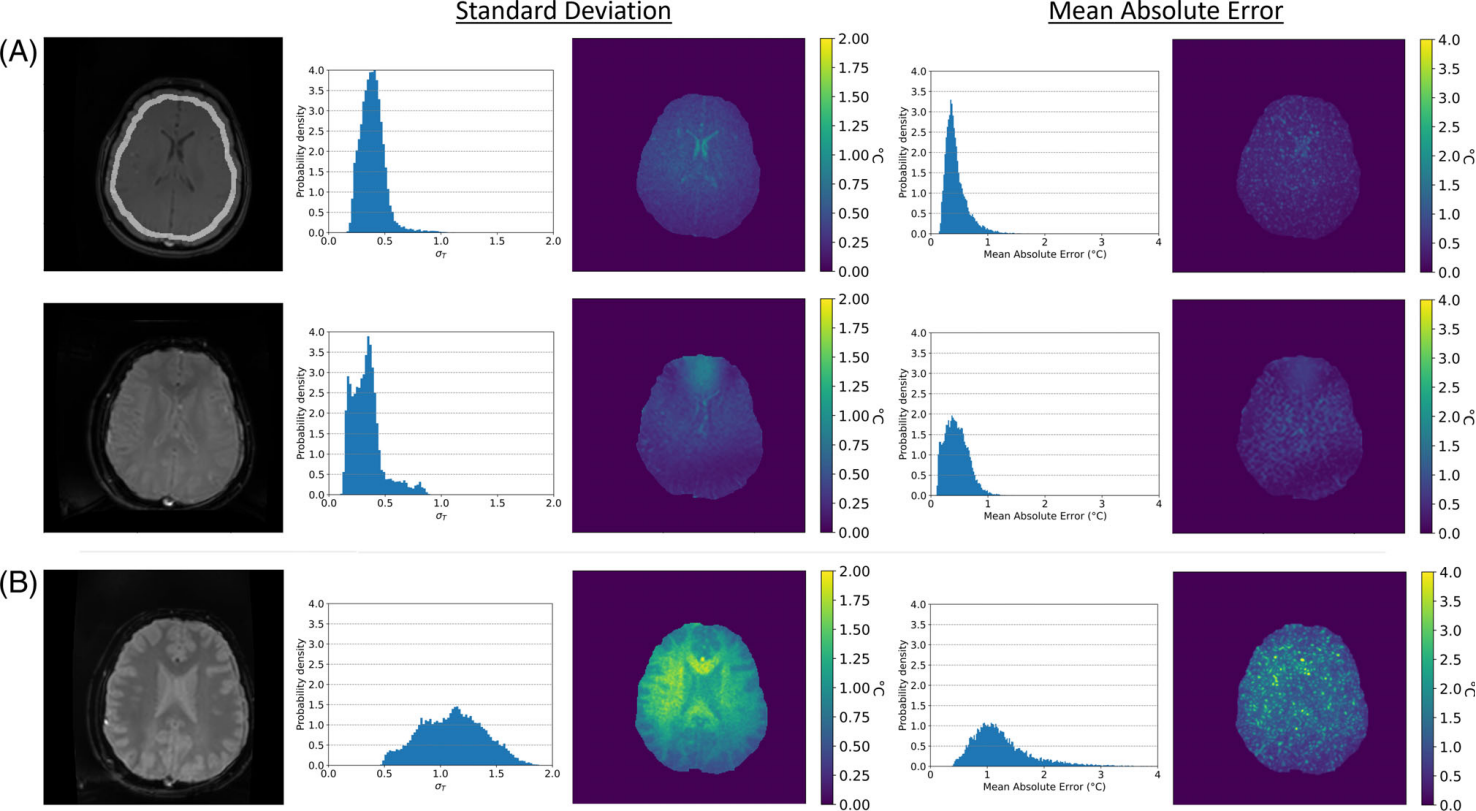
(A) Comparison of a SE‐GRE temperature mapping (top row), vs. a SS‐EPI thermometry approach (second row). Both sequences measured over a set of 64 acquisitions, with parameters shown in Table 1. Structural scans shown on the left. A gray band in the first scan shows the region used for background phase removal. In the middle, a SD over the 64 acquisitions is shown as a histogram and SD map. The SE‐GRE average temperature SD over the slice was 0.40°C with a peak deviation of 1.0°C in the brain stem. In the EPI case, temperature SD over the slice averaged 0.33°C with a peak deviation of 0.90°C. The right shows the MAE over the 64 acquisitions, with the histogram of the MAE and a map of the MAE. The SE‐GRE approach had an average MAE of 0.45°C and the single‐shot three‐slice EPI had an average MAE of 0.45°C. (B) Structural (left), SD histogram and map (middle), and MAE histogram and map (right) measured using 64 EPI acquisitions over a larger coverage of 13 slices, with parameters shown in Table 1. With a 1.5 s update rate, temperature SD over the slice averaged 1.2°C with a peak SD of 2.1°C. The MAE over this sequence averaged 1.3°C. Structure can more clearly be seen in the temperature stability figure; more exact temperature mapping possible in the gray matter.

In‐vivo temperature stability measurements for each protocol are shown in Figure 4. The mean and peak temperature time series SDs of the SE‐GRE acquisition were measured at 0.40°C (with a SD on this measurement of 0.11°C) and 1.0°C, while the MAE analysis reported an average of 0.45°C, with 15 547 voxels in the masked region. The mean and peak temperature SD of the averaged ss‐EPI acquisition was measured at 0.34°C (with a SD on this measurement of 0.14°C) and 0.9°C with 15 745 voxels masked. The MAE of this protocol had an average of 0.45°C.

The SE‐GRE and averaged ss‐EPI were compared using an unpaired t‐test, which showed a statistically significant difference in the SDs between the two protocols (*p* < 0.0001). When comparing the MAE in both, the result was found to be not statistically significant (*p* = 0.0179 > 0.01).

The mean and peak temperature SDs of the ss‐EPI acquisition were measured at 1.15°C (with a SD on this measurement of 0.28°C) and 2.05°C. The MAE analysis had an average of 1.26°C. SE‐GRE and EPI acquisitions have substantially different contrasts which result in temperature precision differences between in CSF, gray matter, and white matter.

## 4 DISCUSSION

Production of the two‐cylinder phantom for use in thermal tracking provided benefits compared to a more common doped water bath approach seen in many thermometry phantom experiments. Having two regions thermally separated by the foam layer between allows for a stable reference temperature contained within the imaging region which can be measured/tested and monitored with a reference temperature probe. The design of the heated gel tube in the molded foam allows for quick subsequent temperature mapping experiments with little downtime as tubes can be quickly changed. One of the strengths of using HEC is a high viscosity, as the gelled saline limits the convective transport of heat. This offers the potential ability to measure heat accumulation of thermal therapies in the phantom gel.

A first‐order background phase removal was used before phase unwrapping was performed. It was found in our experience that a 0th‐order was not sufficient to remove environmental and system background phase instabilities. For example, when using first‐order background phase removal over 0th order, all methods showed reductions in MAE within the reference region: 1.3°C to 0.6°C (SE‐GRE), 0.7°C to 0.2°C (averaged ss‐EPI), and 1.2°C to 0.4°C (ss‐EPI). Linear correction was found to be sufficient, with previous work having shown corrections of fourth‐order being used,^12^ along with first‐order corrections being typical.^26^ In practice, for applications where there is no available non‐heated reference region (e.g., hyperthermia treatment), reference vials can be used.

Both the temperature tracking experiment and the in vivo stability measurements at 0.5T showed good performance using a SE‐GRE PRF thermometry approach. Given that the update rate and temperature precision is sufficient,^27^ a single‐shot approach can be used to reduce the update rate while not introducing distortions. A notable leap in performance when using the averaged ss‐EPI was observed, where the tracking SD was statistically significantly improved at a similar update rate. As seen in Figures 1 and 2, the EPI method provided a smaller spread of measurement bias in tracking temperature changes when comparing measurements to a fiber optic temperature probe. Should the 7.6 s update rate provided by these two protocols be acceptable, the EPI approach likely provides a better option for PRF thermometry. The SE‐GRE acquisition employed an TE of 23.75 ms, which is substantially lower than the optimal TE of approximately 90 ms. The use of optimal TEs for this acquisition would result in prohibitively slow uprate rates.

In many applications, larger spatial coverage is desired. The efficient EPI acquisition scheme offers opportunities to trade‐off temporal resolution for spatial coverage. This trade‐off was explored using the non‐averaged ss‐EPI acquisition, which provided a lower SD during the cooling experiment than the SE‐GRE acquisition while exhibiting an update rate over five times faster. In vivo, the ss‐EPI exhibited worse SD and MAE, however this trade off may be worthwhile if the improvement in update rate and number of slices is of interest.

All temperature measurements showed adequate temperature precision (˜1°C)^27^ both in phantoms and in vivo. The averaged ss‐EPI acquisition offers lower temperature SD than the SE‐GRE acquisition. This improvement was most pronounced in the phantom cooling experiment where the temperature accuracies were improved by 50% using EPI matched to the same time. The improvement was less pronounced in the in vivo stability measurements where mean temperature precision improved by 18%, and it was not possible to conclude a better approach based on the mean absolute error in vivo. This reduced improvement is likely caused by physiological effects such as breathing and subject motion which would be most pronounced in regions with high susceptibility. These regions would likely benefit from an TE shorter than 70 ms due to decreased $T_{2}^{*}$. A multi‐echo EPI acquisition would likely mitigate this effect.^28^

In some of the cooling experiments, a subtle mismatch was observed between the temperature probe and the measurements. It is unclear what the exact source of the discrepancy is. The drift could be from an external (spatially second‐order or higher) source,^29^ deviations between the true probe location and measured values, and/or spatial variations within the measurement region. Unfortunately, the phantom design used in this work limited our ability to test higher spatial order background phase removal, as post masking the reference region was relatively small. Additionally, previous studies have shown that gradient coil heating^30^ over time can impact thermometry, especially in high‐duty cycle acquisitions such as EPI. In this work, efforts were taken to reduce this effect by running dummy scans prior to cooling experiments. However, it is important to note that after reduction efforts and first‐order correction, the observed drift is much smaller than practical precision values required for thermal therapies and are comparable in magnitude to the precision in the ground truth temperature probe.

## 5 CONCLUSIONS

Temperature mapping using PRF‐based approaches on a mid‐field cryogen‐free MR scanner using both single echo GRE and EPI were compared. Using two comparisons, an averaged single‐shot EPI approach was shown to outperform the SE‐GRE PRF, as in vivo the EPI‐based approach showed better temperature SDs in the brain when both methods used similar acquisition times. Further, when comparing temperature tracking in a phantom using a Bland–Altman plot, the temperature measurement bias spread for the EPI implementation was smaller than when tracked using the single echo method, with a statistically significant improvement in the tracking precision. Also, an optimized unaveraged single‐shot EPI sequence matched the SE‐GRE temperature precision while performing a cooling experiment while providing a 5× faster update rate.
